# Pharmacokinetic Alteration of Baclofen by Multiple Oral Administration of Herbal Medicines in Rats

**DOI:** 10.1155/2014/402126

**Published:** 2014-10-29

**Authors:** Tae Hwan Kim, Gi-Young Park, Soyoung Shin, Dong Rak Kwon, Won Sik Seo, Jeong Cheol Shin, Jin Ho Choi, Sang Hoon Joo, Kwon-Yeon Weon, Byung Sun Min, Kyung Min Baek, Mahesh Upadhyay, Bing Tian Zhao, Mi Hee Woo, So Hee Kwon, Beom Soo Shin

**Affiliations:** ^1^School of Pharmacy, Sungkyunkwan University, Suwon, Gyeonggi-do 440-746, Republic of Korea; ^2^Department of Rehabilitation Medicine, School of Medicine, Catholic University of Daegu, Daegu 705-718, Republic of Korea; ^3^Department of Pharmacy, College of Pharmacy, Wonkwang University, Iksan, Jeonbuk 570-749, Republic of Korea; ^4^College of Pharmacy, Catholic University of Daegu, 13-13 Hayang-ro, Hayang-eup, Gyeongsan-si, Gyeongbuk 712-702, Republic of Korea; ^5^Department of Cardiovascular and Neurologic Diseases, College of Oriental Medicine, Daegu Haany University, Daegu 706-060, Republic of Korea; ^6^College of Pharmacy, Yonsei Institute of Pharmaceutical Sciences, Yonsei University, Incheon 406-840, Republic of Korea

## Abstract

The potential pharmacokinetic (PK) interaction of conventional western drug, baclofen, and oriental medications Oyaksungisan (OY) and *Achyranthes bidentata radix* (AB) extract for the treatment of spasticity has been evaluated. Rats were pretreated with distilled water (DW), OY, or AB extract by oral administration every day for 7 days. After 10 min of the final dose of DW or each herbal medication, baclofen (1 mg/kg) was given by oral administration and plasma concentrations of baclofen were determined by LC/MS/MS. The plasma baclofen concentration-time profiles were then analyzed by noncompartmental analysis and a population PK model was developed. Baclofen was rapidly absorbed, showed biexponential decline with elimination half-life of 3.42–4.10 hr, and mostly excreted into urine. The PK of baclofen was not affected by AB extract pretreatment. However, significantly lower maximum plasma concentration (*C*_max_) and longer time to reach *C*_max_ (*T*_max_) were observed in OY pretreated rats without changes in the area under the curve (AUC) and the fraction excreted into urine (*F*_urine_). The absorption rate (*K*_*a*_) of baclofen was significantly decreased in OY pretreated rats. These data suggested that repeated doses of OY might delay the absorption of baclofen without changes in extent of absorption, which needs further evaluation for clinical significance.

## 1. Introduction

Spasticity is a muscle control disorder characterized by an abnormal increase in muscle tone or muscle stiffness due to the impaired nerve pathways. It may occur in association with cerebral palsy, traumatic brain injury, stroke, multiple sclerosis, spinal cord injury, and so forth. Symptoms of spasticity may include abnormal posture, carrying the shoulder, arm, wrist, and finger at an abnormal angle, exaggerated deep tendon reflexes, repetitive jerky motions, scissoring, and fixed joints [1], which may significantly interfere with patients' daily activities.

Baclofen (Lioresal), the first synthesized structural analogue of *γ*-aminobutyric acid (GABA), has been used as the reference treatment for spasticity. Other management options such as therapeutic exercise, physical modalities, complementary/alternative medicine interventions, and chemodenervation are also available [2, 3].

A variety of herbs are another option for treating spasticity which can help alleviate tight and sore muscles. Oyaksungisan (OY) is a traditional prescription of oriental medicine which is a water extract of twelve herbs including ephedra herb,* Citrus unshiu* peel, lindera root, cnidii rhizoma,* Angelica dahurica* root,* Bombyx batryticatus*, Aurantii fructus immaturus,* Platycodon* root, Zingiberis rhizoma, Glycyrrhizae radix et rhizoma, Zingiberis rhizoma crudus, and Zizyphi fructus [4]. OY has been used for treatment of paralysis and rheumatoid arthritis [5] and approved as an over-the-counter drug in Korea for the treatment of paralysis and muscle pain. Recent studies demonstrated that OY also possesses neuroprotective activity, anti-inflammation effect [5], and anticancer activity on human colon cancer cells [4].* Achyranthes bidentata* radix (AB) is another traditional medicinal plant which has been used for spasm. AB is derived from the roots of* Achyranthes bidentata* Blume and* Achyranthes japonica* Nakai in the Korean Pharmacopeia,* A. bidentata* Blume in the Chinese Pharmacopoeia, and* A. bidentata* Blume and* A. fauriei* Leveille et Vaniot in the Japanese Pharmacopoeia [6]. AB has been commonly prescribed not only for spasticity but also for treating of various conditions including stroke by promoting the blood circulation and stasis removal [7, 8], arthritis, hypertension, and so forth [9]. It has also been reported that AB possesses neuroprotective [7], cardioprotective [10], and anti-inflammatory [11] effects in experimental animals.

These herbal medicines are often used as self-medication and available as over-the-counter drugs without prescription. Thus, they may be used in combination with conventional drugs, and the potential of their interactions is raising concern [12]. Concurrent use of the herbs and pharmaceutical drugs may mimic, augment, or oppose the pharmacokinetic (PK) or pharmacodynamic (PD) properties thereby increasing or decreasing the pharmacological or toxicological effects of either constituent. Since the PK and PD characteristics of most of the herbal drugs are not completely known, probable drug-herb interactions are not easily anticipated [13, 14].

Therefore, we aimed to evaluate the potential PK interaction of baclofen with OY and AB, which are commonly used for the treatment of spasticity. Our study examined the effects of OY and AB extract on baclofen PK in rats by evaluating the changes in baclofen PK following multiple oral administration of each herbal medicine. The noncompartmental PK parameters were assessed and a population PK model was developed to describe and evaluate the alterations of baclofen PK.

## 2. Materials and Methods

### 2.1. Materials

Baclofen, gabapentin (internal standard, IS), glycyrrhizin, hesperidin, and ferulic acid were purchased from Tokyo Chemical Industry Co., Ltd. (Tokyo, Japan). Naringin and 6-Gingerol were purchased from Sigma-Aldrich Co. LLC. (St. Louis, MO, USA). Neohesperidin was purchased from Santa Cruz Biotechnology, Inc. (Dallas, Texas, USA). Ecdysterone and R,S-Inokosterone were obtained from analytical chemistry laboratory in College of Pharmacy at Catholic University of Daegu. Acetonitrile, methanol, and distilled water (all HPLC grades) were purchased from Mallinkrodt Baker, Inc. (Phillipsburg, NJ, USA). Perchloric acid and formic acid were purchased from Aldrich Chemical Co. (Milwaukee, WI, USA). Oyaksungisan Extract Granule was obtained from Hankook Shinyak Corp. (Nonsan, Chungnam, Korea).* Achyranthes bidentata *radix was obtained from Daegu Haany University (Gyeongsan, Gyeongbuk, Korea).

### 2.2. Preparation of AB Extract and Determination of Standard Constituents in AB Extract and OY

Extraction of AB was performed in accordance with the Notification number 2003-17 of the Ministry of Food and Drug Safety, Korea (2003). Herbal materials were crushed and separated by passing through a standard #35 sieve (500 *μ*m) to obtain homogeneous particles. The obtained herbal specimen was then extracted in distilled water (10 times of the volume of the initial weights of the herbal specimen) at 100°C for 2-3 hours and filtered out. Following evaporation of the remaining water by using a rotary evaporator, the final herbal extracts were obtained by freezing at −80°C and lyophilization. The extraction yield of AB extract was 30.4%.

To guarantee the consistent quality of the herbal formula extract, the constituents of AB extract and OY were determined by HPLC coupled with UV detector [6] and diode array detector [4], respectively. The analysis conditions were summarized in Supplementary Table 1  available online at http://dx.doi.org/10.1155/2014/402126. The standard composition for AB extract is Ecdysterone, R-Inokosterone, and S-Inokosterone and that of Oyaksungisan (OY) is glycyrrhizin, naringin, hesperidin, and neohesperidin; 6-Gingerol and ferulic acid were selected based on the literature [4, 6, 15] and database provided by Korea Institute of Oriental Medicine (KIOM) [16]. All the retention times of the standard compounds for OY and AB extract were comparable with those from the literature [4, 6]. The HPLC finger print chromatograms for OY and AB extract were shown in Supplementary Figures 1 and 2, respectively, and the contents of the major compounds were presented in Supplementary Table 2.

### 2.3. Animal Study

The animal study was approved by the Ethics Committee for the Treatment of Laboratory Animals at the Catholic University of Daegu (IACUC 2012-11) and conducted following the standard operating procedures (SOPs). Male Sprague-Dawley rats (8-9 weeks, 280–300 g; Hyochang Science, Daegu, Korea) were kept in plastic cages with free access to standard diet (Daejong, Seoul, Korea). The animals were maintained at a temperature of 23 ± 2°C with a 12 hr light-dark cycle and relative humidity of 50 ± 10%.

Distilled water (DW, *n* = 6) or herbal medicine, that is, OY (500 mg/head/day, *n* = 4) and AB extract (130 mg/head/day, *n* = 5), was administered orally for 7 consecutive days. The doses of OY and AB extract were selected based on the human doses and animal toxicity studies. Considering fast metabolic processes of rats compared to human, 8–10-fold higher doses than clinically recommended doses of 15 g/day (=214.3 mg/kg/day) for OY and 10 g/kg/day (=142.8 mg/kg/day as AB) for AB extract were administered. The AB extract dose was calculated considering the yield of AB extract (30.4%). Based on the literature, the no-observable-adverse-effect level (NOAEL) was considered over 5000 mg/kg in mice for OY [17] and 2000 mg/kg in F344 rats for AB [18]. At 10 min after the last dose of DW or herbal medicine, baclofen solution (0.5 mg/mL in distilled water) was given by oral gavage at a dose of 1 mg/kg. Rats were fasted 12 hours prior to the baclofen dose. Approximately 400 *μ*L of the venous blood samples was collected at 5, 15, and 30 min and 1, 2, 4, 8, 12, and 24 hr after dose from jugular vein. Plasma samples were harvested by centrifugation of the blood samples at 16,060 g for 10 min and stored at −20°C until analysis. Urine sample was collected for 24 hr.

### 2.4. LC/MS/MS

Baclofen concentrations were determined by the LC/MS/MS assay as reported previously [19]. Briefly, the internal standard (IS) working solution (4 *μ*L of 200 ng/mL IS solution for plasma samples and 8 *μ*L of 200 ng/mL IS solution for urine samples) and diluted perchloric acid (4 *μ*L of 2 M solution for plasma samples and 40 *μ*L 0.2 M solution for urine samples) were added to 20 *μ*L of each sample as precipitation solvent. The mixture was vortexed and centrifuged for 10 min at 16,060 g. The supernatant was collected and 2 *μ*L of the sample was injected onto the LC/MS/MS.

LC/MS/MS system consisted of API 4000 triple quadrupole mass spectrometer (AB MDS Sciex, Toronto, ON, Canada) coupled with an Agilent 1100 HPLC system (Agilent, Santa Clara, CA, USA). Plasma samples were separated on a Kinetex *C*_18_ column (50 × 2.10 mm I.d., 2.6 *μ*m) with a KrundKatcher ultra column inline filter (Phenomenex, Torrance, CA, USA) and the mobile phase was composed of a mixture of acetonitrile and 0.05% of formic acid (10 : 90 v/v). Urine samples were separated on Agilent Zorbax SB-Aquasil Column (100 × 2.10 mm I.d., 3.5 *μ*m; Agilent, Santa Clara, CA, USA) with SecurityGuard Cartridge (Phenomenex, Torrance, CA, USA) and the mobile phase was a mixture of acetonitrile and 0.05% of formic acid (5 : 95 v/v). The flow rate was set at 0.2 mL/min and the column oven temperature was 30°C for all samples. The electrospray ionization (ESI) source was operated in positive mode with the curtain and turbo-gas (all nitrogen) set at 20 and 6 psi, respectively. The turbo-gas temperature and the ion spray needle voltage were set at 450°C and 4500 V, respectively. The mass spectrometer was operated at unit resolution for the first quadrupole (Q1) and low resolution for the third quadrupole (Q3) in the multiple reaction monitoring (MRM) mode with a dwell time of 300 ms per MRM channel. The selected precursor/product ion pairs were* m/z* 214.3 → 151.1 for baclofen and* m/z* 172.2 → 154.2 for IS. The collision energy was set at 23 and 19 eV for baclofen and IS, respectively. Data acquisition was performed with Analyst 1.4 software (AB MSD Sciex, Toronto, Canada).

The assay was linear over a concentration range of 0.25–500 ng/mL for rat plasma and 2–5000 ng/mL for urine with correlation coefficients >0.999. The mean intra- and interday assay accuracies were 94.6–104.6 and 96.0–103.6%, respectively. The mean intra- and interday precisions were ≤5.71 and ≤5.70%, respectively.

### 2.5. Noncompartmental Analysis

For descriptive PK analysis, PK parameters were determined by the noncompartmental analysis using WinNonlin (version 2.1; Pharsight, Cary, NC, USA). These parameters included terminal half-life (*t*_1/2_), area under the serum concentration-time curve from time zero to the last observation time point (AUC_all_) and infinity (AUC_inf⁡_), apparent volume of distribution during the terminal phase (*V*_*z*_), systemic clearance (CL), and fraction of dose excreted into urine (*F*_*e*,urine_). The peak serum concentration (*C*_max⁡_) and the time to reach *C*_max⁡_ (*T*_max⁡_) were read directly from the observations. The absolute bioavailability (*F*) of baclofen after oral dosing was calculated as *F* = (Dose_iv_ · AUC_oral_)/(Dose_oral_ · AUC_iv_), where AUC_iv_ was obtained from the previous study [19].

### 2.6. Population PK Modeling

The structural model was designed to capture the absorption, disposition, and elimination of baclofen. We considered a PK model with two compartments and first-order absorption of baclofen. The absorption process after oral administration of baclofen was described as a first-order rate constant, *K*_*a*_. The differential equation for the amount of drug in the absorption site (*X*_gut_) was

(1) $$\begin{array}{c} \frac{\text{d}X_{\text{gut}}}{\text{d}t}=-K_{a}\cdot X_{\text{gut}}. \end{array}$$

Bioavailability of baclofen was calculated by using the internal function of SADAPT, BOLUSF which calculates the relative fraction of the dose to enter into the systemic absorption site after oral administration comparing to that after intravenous injection. With the bioavailability of BOLUSF_control_, BOLUSF_OY_, and BOLUSF_AB_, the initial condition for *X*_gut_ was BOLUSF_control_ · Dose for the DW, BOLUSF_OY_ · Dose for the OY, and BOLUSF_AB_ · Dose for the AB extract pretreated group. The absorption rates for DW, OY, and AB extract pretreated group, that is, *K*_*a*,control_, *K*_*a*,OY_, and *K*_*a*,AB_, respectively, were estimated separately. The central compartment received drug from the gut compartment. The differential equation for the amount of drug in the central compartment (*X*_1_) contained terms for the drug transfer rates into and from the peripheral compartment and for the drug elimination rates into the urine (*K*_el,ur_) and other processes (*K*_el_) (initial condition: 0):

(2) dX1dt  =Ka·Xgut −(K12+Kel,ur+Kel)·X1+K21·X2.

The *K*_12_ and *K*_21_ are the intercompartmental rate constants between the central (*X*_1_) and peripheral (*X*_2_) compartments for baclofen. The differential equation for the peripheral compartment was (initial condition: 0)

(3) $$\begin{array}{c} \frac{\text{d}X_{2}}{\text{d}t}=K_{12}\cdot X_{1}-K_{21}\cdot X_{2}. \end{array}$$

The differential equation for the urinary excretion was (initial condition: 0)

(4) $$\begin{array}{c} \frac{\text{d}X_{u}}{\text{d}t}=\;K_{\text{el},\text{ur}}\cdot X_{1}. \end{array}$$

Since there is no outward flux from the urine compartment, urinary excretion of baclofen was modeled as the accumulated amount of excreted baclofen via urine.

All plasma concentrations were simultaneously fitted by the population pharmacokinetic (POP-PK) modeling using the importance sampling version of the Monte Carlo parametric expectation maximization (MC-PEM) algorithm in the parallelized S-ADAPT software (version 1.57) [20] supported by the SADAPT-TRAN facilitator [21, 22]. Log-normal distribution was used to describe the between-subject variability (BSV) for each parameter. Residual model with additive and proportional error was used for baclofen concentrations [21]. The goodness of fit was assessed by visual inspection of the observed and fitted concentrations, the objective function, plausibility of parameter estimates, standard diagnostic plots, the normalized prediction distribution error (NPDE) [23], and visual predictive checks (VPCs) [24].

### 2.7. Statistical Analysis

The obtained parameters were compared by unpaired *t*-test between the two means for unpaired data or one-way ANOVA followed by Scheffe's post hoc test among more than two means for unpaired data. *P* values < 0.05 were considered as statistically significant.

## 3. Results

### 3.1. Pharmacokinetics of Baclofen after Multiple Doses of OY and AB Extract

**Figure 1:**
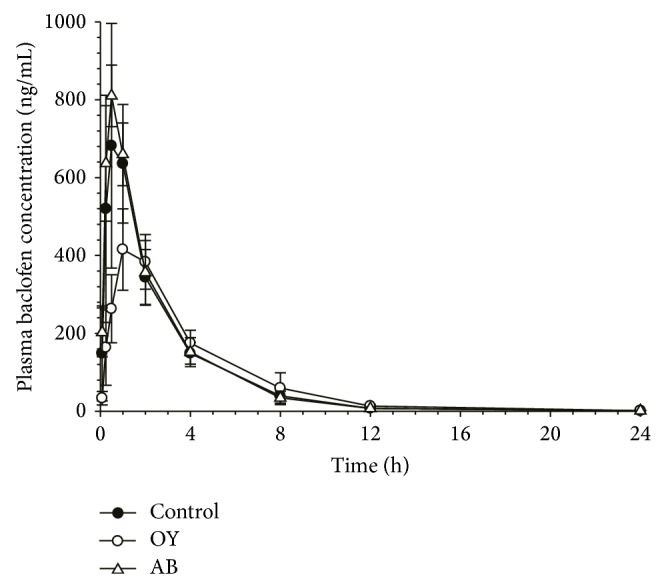
Average plasma concentration-time profiles of baclofen obtained after oral administration of baclofen (1 mg/kg) to rats pretreated with distilled water (control), Oyaksungisan (OY), or* Achyranthes bidentata* radix (AB) extract. Distilled water or each herbal medicine was administered orally for 7 consecutive days and baclofen was administered after 10 min of distilled water or each herbal medicine on the final day.

**Figure 2:**
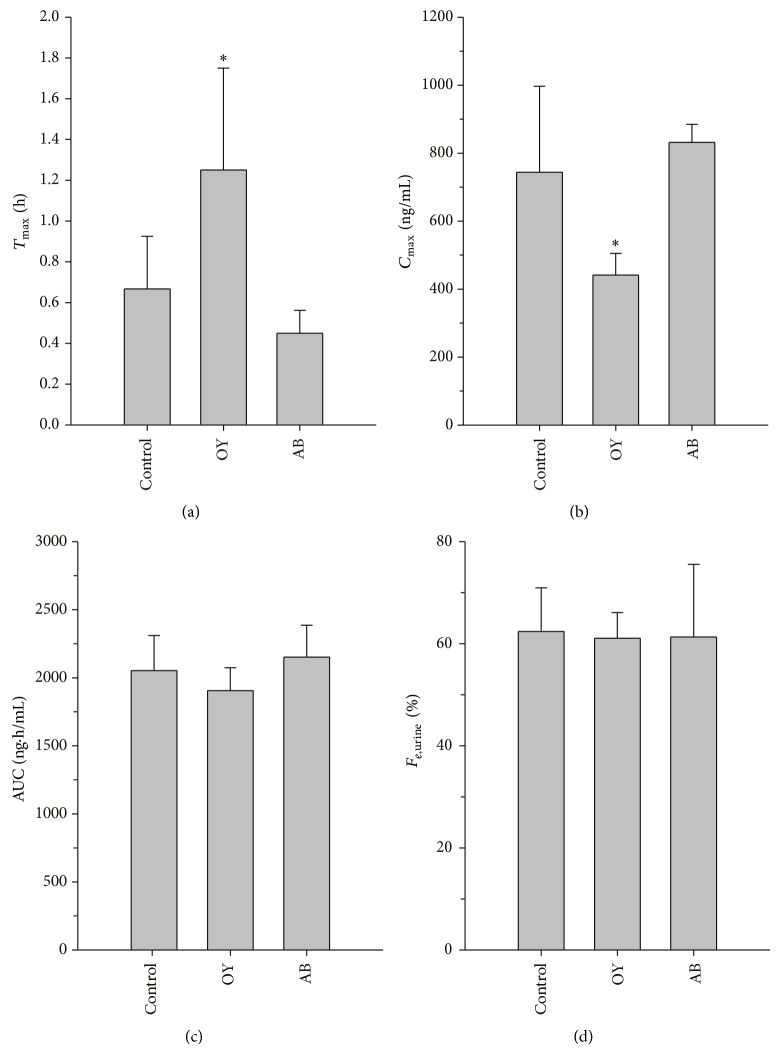
Noncompartmental pharmacokinetic parameters (a) *T*_max⁡_, (b) *C*_max⁡_, (c) AUC, and (d) *F*_*e*,urine_ of baclofen obtained after oral administration of baclofen (1 mg/kg) to rats pretreated with distilled water (control), Oyaksungisan (OY), or* Achyranthes bidentata* radix (AB) extract. Distilled water or each herbal medicine was administered orally for 7 consecutive days and baclofen was administered after 10 min of distilled water or each herbal medicine on the final day.

To examine the effects of herbal medicines on PK of baclofen, the herbal medicines were administered daily for 7 successive days prior to administration of baclofen and baclofen PK of control and test groups was compared. In control group, DW was administered daily for 7 successive days. No significant changes in the baclofen PK were observed after administration of DW for 7 days compared to the results of our previous study which was conducted using intact rats without any pretreatment [14]. The average plasma concentration-time profiles of baclofen obtained after oral administration of baclofen (1 mg/kg) to rats pretreated with DW, OY, or AB extract for 7 days are presented in Figure 1. The average noncompartmental PK parameters of baclofen were summarized in Table 1. Plasma concentration versus time profiles of control and AB extract treated rats were well comparable. Plasma baclofen increased rapidly after oral administration, reached its maximum concentration (*C*_max⁡_) within 1 hour, and declined thereafter. The PK parameters of baclofen following AB extract pretreatment were not significantly different from those of the control group. Although the overall concentration versus time profile looks similar to other groups, baclofen *C*_max⁡_ was found to be significantly lower in OY pretreated rats compared to that in DW pretreated rats (441.50 ± 63.33 versus 744.00 ± 252.96 ng/mL, *P* < 0.05) and the time to reach *C*_max⁡_ (*T*_max⁡_) was also longer in OY pretreated rats (1.25 ± 0.50 versus 0.67 ± 0.26 hr, *P* < 0.05). Nevertheless, all other PK parameters including AUC and *F*_*e*,urine_ after OY pretreatment were similar to control (Figure 2).

### 3.2. Population Pharmacokinetic Modeling

**Figure 3:**
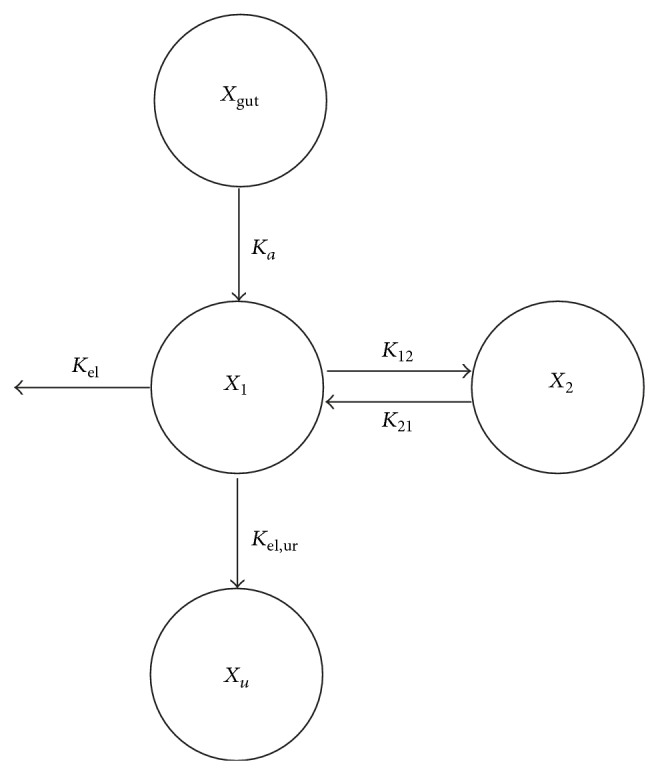
Schematic diagram of the pharmacokinetic model for baclofen. The model consisted of two compartments and first-order absorption of baclofen. *X*_gut_, *X*_1_, *X*_2_, and *X*_urine_ = amount of drug in the gut, central, peripheral, and urine compartments, respectively; *K*_*a*_ = first-order absorption rate constant; *K*_12_ and *K*_21_ = intercompartmental rate constants between the central (*X*_1_) and peripheral (*X*_2_) compartments for baclofen; *K*_el,ur_ and *K*_el_ = drug elimination rates into the urine and other processes, respectively.

**Figure 4:**
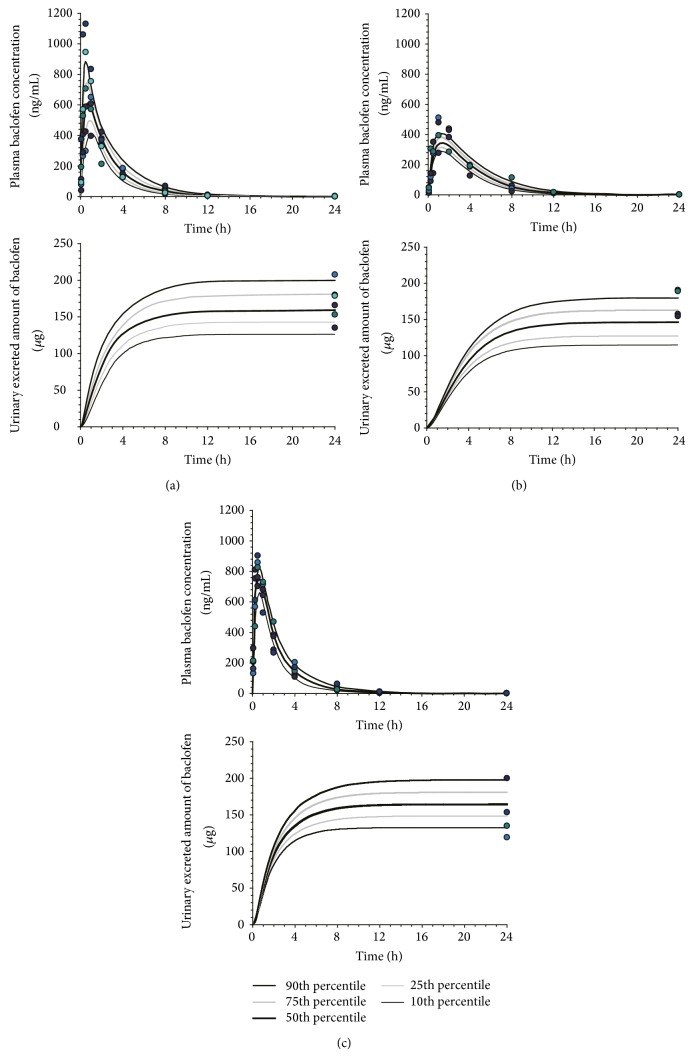
Visual predictive check of the pharmacokinetic model for baclofen. The observed plasma concentration and urinary excretion data after oral administration of baclofen (1 mg/kg) to rats pretreated with (a) distilled water (control), (b) Oyaksungisan (OY), or* Achyranthes bidentata* radix (AB) extract (closed circles) are shown with lines representing the median population predictions and 10th, 25th, 75th, and 90th percentiles according to the pharmacokinetic model described in Figure 3.

The proposed model contained 2 disposition compartments and a gastrointestinal compartment for baclofen (Figure 3). The visual predictive checks by comparing predicted versus observed plots showed an excellent predictive performance for oral administration of the POP-PK model (Figure 4). The POP-PK parameter estimates for baclofen following oral administration of each pretreatment group are summarized in Table 2. The relative standard errors of all estimated population means were below 23.1%.

The absorption of the orally administered baclofen was adequately described by a first-order process. The estimated bioavailabilities of baclofen after repeated doses of DW, OY, and AB extract by POP-PK modeling were similar to each other and well comparable to those obtained by noncompartmental analysis (Tables 1 and 2). Consistent with the results from noncompartmental analysis, the absorption rate constant of baclofen in OY pretreatment group (*K*_*a*,OY_) was estimated to be lower than that in DW (*K*_*a*,control_) or in AB extract pretreatment group (*K*_*a*,AB_), suggesting slower absorption of baclofen after repeated doses of OY (Table 2).

The estimated urinary excretion of baclofen after repeated doses of DW, OY, and AB extract by the POP-PK model showed good agreement with observed values (Figure 4).

## 4. Discussion

In the present study, potential drug-drug interaction between baclofen and traditional herbal medicines, OY and AB extract was examined. The effects of OY and AB extract on the baclofen PK were analyzed by means of noncompartmental analysis and further assessed by POP-PK modeling approach.

Baclofen was rapidly absorbed after oral administration and showed biexponential decline with the elimination half-life of 3.42–4.10 hr in rats and significant amount of baclofen was found to be excreted into urine as an unchanged form. The PK characteristics of baclofen observed in this study were well comparable with the previous reports [19, 25–27].

Our data clearly suggested that the absorption of baclofen might be significantly delayed by repeated doses of OY (Figure 2). The noncompartmental PK parameters which reflect the absorption rates, that is, *T*_max⁡_ and *C*_max⁡_, were significantly lower following OY pretreatment compared to the control group. However, the extent of absorption of baclofen, represented by the area under the curve (AUC) and the fraction excreted into urine (*F*_urine_), was not affected by OY administration (Figure 2).

These findings were further analyzed by a POP-PK modeling to quantitatively evaluate the alterations in baclofen PK. POP-PK analysis is known to be valuable to investigate potential drug-drug or drug-herb interactions [28]. The predicated concentration versus time profiles of baclofen in plasma and urine showed good agreement with observed profiles (Figure 4). The validity of the model was also demonstrated by excellent agreement between the observed values and the PK parameter estimates, that is, relative error between observed and estimated bioavailability being less than 3.63%. The POP-PK analysis strongly suggested that the absorption process (represented by the rate constant, *K*_*a*_) was significantly delayed, while the bioavailability of baclofen in OY pretreatment group was comparable with that in the control group. Based on the estimated *K*_*a*_ values for each treatment group, baclofen was expected to take more than twice of time to be absorbed after OY dose compared to control (absorption half-life = 1.33 hr versus 0.54 hr) and to achieve lower peak concentration.

The slower rate of absorption of baclofen following repeated doses of OY may be associated with changes in gastrointestinal physiology, such as increased gastric pH, reduced intestinal blood flow, and diminished gastrointestinal motility. OY consists of twelve herbs and some of the components are known to have gastrointestinal effects. Among these, ephedra is well known to diminish gastrointestinal motility [29], which may in part contribute to the changes in baclofen PK.

The PK alteration of baclofen, that is, slower rate of absorption with similar systemic exposure caused by coadministration of OY, allowed sustained exposure of baclofen as a result. Common side effects of baclofen include drowsiness, dizziness, weakness, confusion, and fatigue. More severe side effects are associated with abrupt withdrawal of baclofen, which include hallucinations, confusion, manic psychotic episodes, seizures, autonomic dysreflexia, hyperthermia, and rebound severe spasticity [30, 31]. These side effects are likely related to the fluctuation of the baclofen concentration in the central nervous system. Consistently, intrathecal baclofen pumps [32, 33] were able to maintain higher cerebrospinal fluid (CSF) concentration of baclofen by continuous release of the drug directly into CSF, thereby, significantly reducing the incidence of adverse effects associated with central nervous system [34]. The sustained systemic exposure of baclofen by OY coadministration may lead to similar benefits by maintaining baclofen concentration in CSF in a sustained manner as well. The clinical significance of the potential benefits of OY on baclofen therapy would be evaluated in further studies.

## 5. Conclusions

The potential pharmacokinetic interaction of conventional western drug and oriental medication for the treatment of spasticity has been evaluated by noncompartmental and POP-PK modeling analysis. Following the repeated doses of OY, sustained systemic exposure of baclofen was observed caused by the slower rate of absorption without alteration in the extent of absorption.

## Supplementary Material

Supplementary Material includes analytical conditions of determination of the representative standard compounds and their contents in Oyaksungisan and Achyranthes bidentata radix extract as well as the fingerprint chromatograms.

## Figures and Tables

**Table 1:** Average noncompartmental pharmacokinetic parameters of baclofen obtained after oral administration of baclofen (1 mg/kg) to each pretreated group. Distilled water (control) or each herbal medicine, that is, Oyaksungisan (OY) or *Achyranthes bidentata* radix (AB) extract, was administered orally for 7 consecutive days. On the final day, baclofen was administered orally after 10 min of the distilled water or each herbal medicine.

Parameters	Control (*n* = 6)	OY (*n* = 4)	AB extract (*n* = 5)
*t*_1/2_ (hr)	4.10 ± 2.52	3.42 ± 0.74	3.64 ± 1.33
*T*_max⁡_ (hr)	0.67 ± 0.26	1.25 ± 0.50^*^	0.45 ± 0.11
*C*_max⁡_ (ng/mL)	744.00 ± 252.96	441.50 ± 63.30^*^	831.80 ± 53.00
AUC_all_ (ng*·*hr/mL)	2042.33 ± 247.68	1899.00 ± 167.91	2144.54 ± 231.19
AUC_inf_ (ng*·*hr/mL)	2051.75 ± 259.74	1905.79 ± 168.62	2152.10 ± 233.63
*V*_*z*_/*F* (L/kg)	2.78 ± 1.32	2.60 ± 0.54	2.41 ± 0.83
CL/*F* (mL/min/kg)	8.23 ± 1.06	8.80 ± 0.78	7.82 ± 0.85
*F*_*e*,urine_ (%)	62.40 ± 8.56	61.08 ± 5.02	61.31 ± 14.25
*F* (%)	88.02 ± 11.14	81.76 ± 7.23	92.33 ± 10.02

^*^ *P* < 0.05 versus control.

**Table 2:** Population pharmacokinetic parameter estimates for baclofen obtained after oral administration of baclofen (1 mg/kg) to each pretreated group. Distilled water (control) or each herbal medicine, that is, Oyaksungisan (OY) or *Achyranthes bidentata* radix (AB) extract, was administered orally for 7 consecutive days. On the final day, baclofen was administered orally after 10 min of the distilled water or each herbal medicine.

Parameters	Estimate (relative standard error)	
	Population mean	CV for between-subject variability
Parameters for systemic disposition		
*K*_12_ (hr^−1^)	0.803 (12.3%)	10.2% (349%)
*K*_21_ (hr^−1^)	0.965 (6.2%)	4.5% (340%)
*K*_el_ (hr^−1^)	0.839 (11.2%)	18.5% (42%)
*K*_el,urine_ (hr^−1^)	0.190 (12.3%)	6.6% (418%)
*V*_*C*_ (L/kg)	0.448 (0.9%)	1.3% (699%)

Parameters for oral absorption		
*F*_control_ (%)	86.7 (13.9%)	7.2% (342%)
*F*_OY_ (%)	78.8 (14.4%)	3.8% (690%)
*F*_AB_ (%)	89.5 (2%)	1.1% (350%)
*K*_*a*,control_ (hr^−1^)	1.28 (23.1%)	55.8% (95%)
*K*_*a*,OY_ (hr^−1^)	0.523 (14.0%)	16.1% (135%)
*K*_*a*,AB_ (hr^−1^)	1.74 (7.6%)	12.2% (317%)
