# Supplementary material for: Pharmacokinetic Alteration of Baclofen by Multiple Oral Administration of Herbal Medicines in Rats
Source: Evid Based Complement Alternat Med. 2014 Oct 29;2014:402126. doi: 10.1155/2014/402126 (PMC4229966; doi:10.1155/2014/402126)

Supplementary Table 1. HPLC/DAD conditions of determination of representative standard compounds in Oyaksungisan (OY) and Achyranthes bidentata radix (AB) extract.

Conditions	OY	AB
HPLC	Waters 2690 Separation module	
Detector	Waters 2996 Photodiode Array Detector (PDA)	
Column	Luna C_{18} column (5 µm, 4.6 mm x 250 mm)	
Mobile phase	0 min, A[a]:B[b] = 80:20<br>10 min, A[a]:B[b] = 80:20<br>15 min, A[a]:B[b] = 65:35<br>30 min, A[a]:B[b] = 50:50<br>40 min, A[a]:B[b] = 35:65<br>60 min, A[a]:B[b] = 30:70	0 - 40 min, C[c]:D[d] = 85:15
Flow rate	1 ml/min	1 ml/min
Column temperature	35° C	40° C
Injection volume	10 µl	20 µl
UV detection wave length	250, 280, 360 nm	245 nm

[a] 0.1% trifluoroacetic acid in water
[b] Methanol
[c] 0.1% formic acid in water
[d] Acetonitrile

Supplementary Table 2. The cotents of representative standard compounds in Oyaksungisan (OY) or Achyranthes bidentata radix (AB) extract.

Herbal medicine	Compound	Concentration (mg/g)* (n=3)
Oyaksungisan granule	Glycyrrhizin	1.075 ± 0.008
	Naringin	0.226 ± 0.002
	Hesperidin	1.112 ± 0.007
	Neohesperidin	0.006 ± 0.000
	6-Gingerol	Below the limit of detection
	Ferulic acid	0.043 ± 0.002
Achyranthes bidentata radix extract	Ecdysterone	0.691 ± 0.023
	R-Inokosterone	0.503 ± 0.052
	S-Inokosterone	0.457 ± 0.009

*The concentration of each component is represented as milligram (mg) of each component in gram (g) of Oyaksungisan (OY) granule or extracted Achyranthes bidentata Radix (AB) powder.

Supplementary Figure 1. (A) Representative standard compounds and (B) HPLC fingerprint chromatograms of Oyaksungisan (OY) granule.

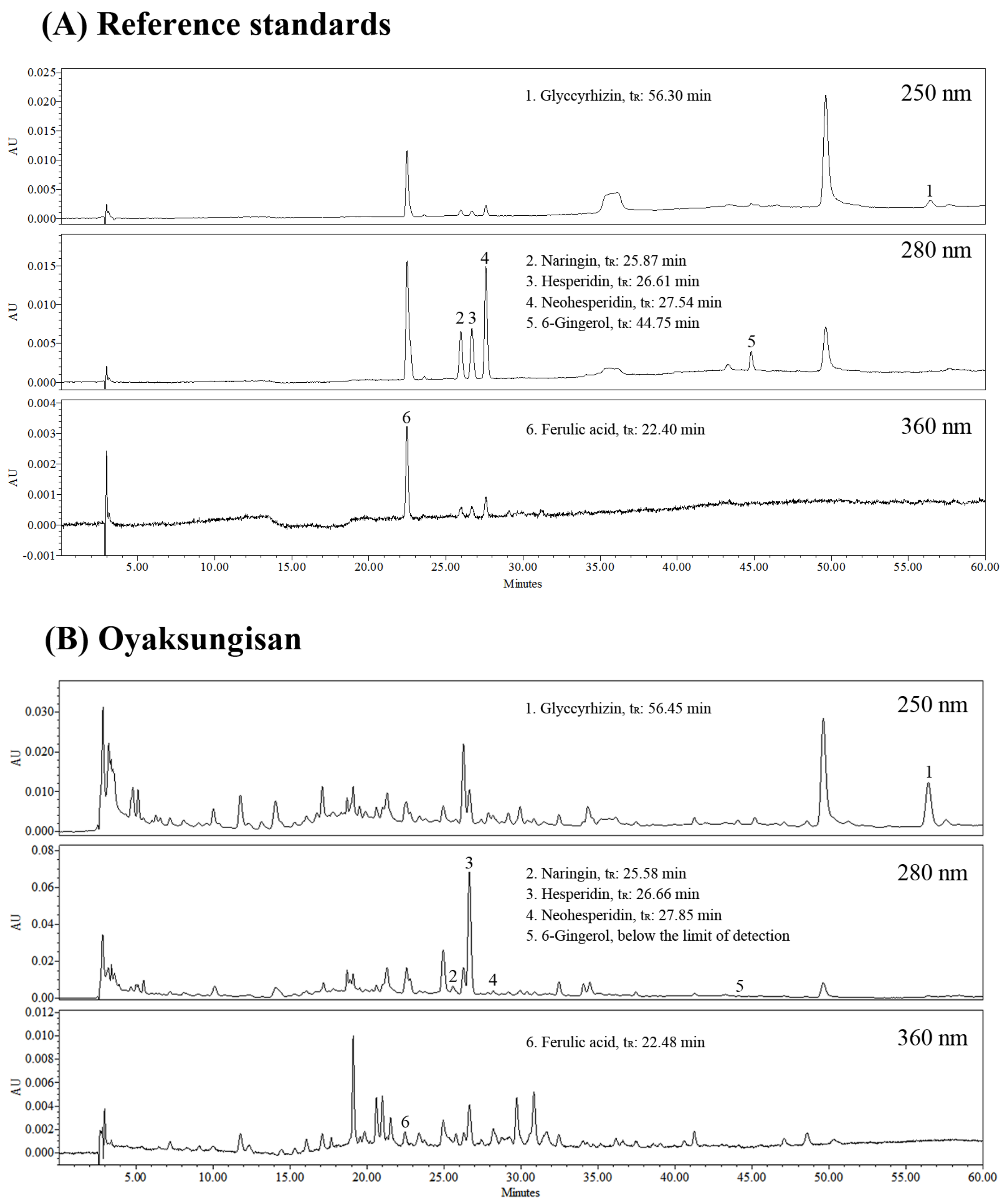

Supplementary Figure 2. (A) Representative standard compounds and (B) HPLC fingerprint chromatograms of Achyranthes bidentata radix (AB) extract.

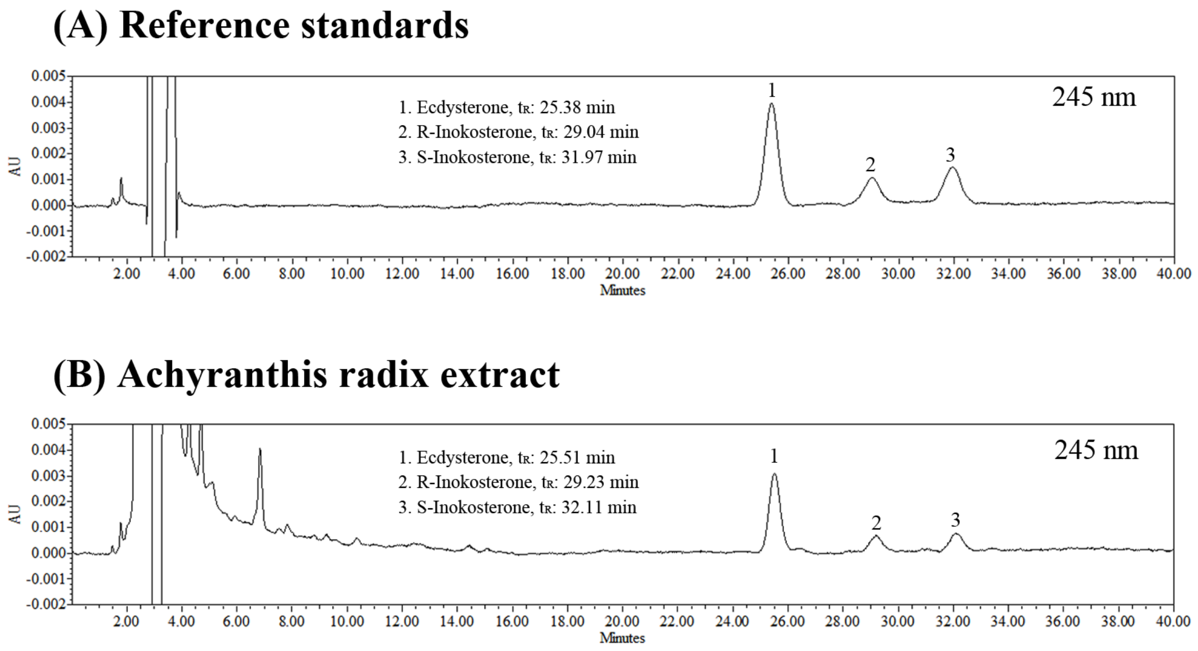

Supplement: Supplementary file 1 — Supplementary Material includes analytical conditions of determination of the representative standard compounds and their contents in Oyaksungisan and Achyranthes bidentata radix extract as well as the fingerprint chromatograms. [file 402126.f1.pdf]
